# What Are the Public Health Effects of Direct-to-Consumer Drug Advertising?

**DOI:** 10.1371/journal.pmed.0030145

**Published:** 2006-03-28

**Authors:** Elizabeth A Almasi, Randall S Stafford, Richard L Kravitz, Peter R Mansfield

## Abstract

Background to the debate: Only two industrialized countries, the United States and New Zealand, allow direct-to-consumer advertising (DTCA) of prescription medicines, although New Zealand is planning a ban [ 1]. The challenge for these governments is ensuring that DTCA is more beneficial than harmful. Proponents of DTCA argue that it helps to inform the public about available treatments and stimulates appropriate use of drugs for high-priority illnesses (such as statin use in people with ischemic heart disease). Critics argue that the information in the adverts is often biased and misleading, and that DTCA raises prescribing costs without net evidence of health benefits.

## Abstract 2

Proponents of such advertising say that it informs the public and stimulates appropriate use of drugs, but critics argue that the adverts are misleading and raise prescribing costs without net evidence of health benefits.

## Elizabeth Almasi and Randall Stafford's Viewpoint: Pharmaceutical Advertising Might Produce a Valuable Placebo Effect

The impact of DTCA on patient expectations has important implications for evaluating its role in the health-care system. While these expectations can lead to inappropriate and excessive prescribing, they also may induce a placebo effect that might increase the clinical effectiveness of the advertised products. This seldom-discussed effect of DTCA should be taken into account in discussion of policy approaches to this form of marketing.

The placebo effect can be triggered by an array of stimuli, such as pills, doctors, and devices. The effect is profound: about one-third of patients report relief from postoperative pain, cough, headache, depression, and other conditions when given a placebo [ 2, 3]. Surprisingly, the two models used to explain the placebo phenomenon are identical to the theories that lie behind the methodologies of consumer advertising.

An enhanced placebo response could improve patient adherence and outcomes.

The first model, classical conditioning, is based on Pavlovian conditioning theory. According to this theory, prior experiences with effective medical treatments “condition” the patient to associate pills, syringes, and authoritative medical opinions with imminent pain relief, eliciting a response similar to the active agents [ 4]. Similarly, DTCA offers conditioned stimuli to associate each product with positive emotions: the joy of playing in beautiful fields for allergy sufferers (loratadine commercial) or the relief conveyed by elderly patients with arthritis participating in their favorite activities (rofecoxib commercial). Patients who take the advertised medication may be conditioned to elicit the positive feelings that were portrayed in the advertisement, which could enhance the medication's clinical effect.

The second theory to explain the placebo effect focuses on the expectancies formed from the information provided [ 5]. According to the expectancy-value theory, individuals are receptive to signals confirming their initial expectancies after administration of a placebo treatment. The ability for information alone to produce a conditioned response explains why patients taking a placebo often report the same side effects as patients taking the active medication: the reported or observed experience of others can elicit a placebo effect by creating the expectancy of an effect. Likewise, many pharmaceutical advertisements teach viewers what to expect from the medication to capitalize on this conditioned response [ 6]. Commercials for conditions such as high cholesterol and osteoporosis first assert that widely prevalent minor symptoms or unassessed biological parameters can have grave implications [ 7]. Then, the promoted drug is introduced as the solution, and the relief associated with the drug is depicted in the advertisement, teaching the viewer what to expect.

These advertising strategies not only create consumer demand for the advertised products, but may also create the emotionally conditioned responses and expectancies instrumental to enhancing a placebo effect that occurs when the medication is taken. This conditioned response may increase the effectiveness of medications beyond that which is expected from their purely biological mechanisms.

Through the placebo effect, patients' positive expectations from DTCA may potentially reduce the amount of treatment requested or required [ 8]. An enhanced placebo response also could improve patient adherence and outcomes [ 9]. To the extent that advertisements “reward” patients for the same actions that physicians recommend, patients may be more likely to follow treatment instructions [ 10]. In addition, physicians may facilitate a placebo response to the medications they prescribe by successfully borrowing strategies from DTCA. In fact, improved communication

Optimal use of DTCA may require stricter guidelines.

might result from personalizing the need for treatment, placing treatment benefits in perspective relative to drug side effects, and providing testimonial examples of past treatment successes. In addition, where a rationale for a class effect exists, physicians may enhance the effect of generic drugs by pointing out their inherent similarities to highly advertised, brand name medications.

Patients' heightened expectations also may motivate them to collaborate with their physicians, and thereby increase the quality of their care. Berger and colleagues suggest that patient expectation and physician perception of patient expectation for prescription medication correlate with the issuance of a prescription [ 11]. Kravitz et al. confirmed this hypothesis. Assuming that the request for a prescription signals a patient's expectation, it is not surprising that in their study, standardized patients suffering from major depressive disorder who requested an antidepressant were much more likely to receive a prescription for antidepressants than patients who made no such request [ 12].

Yet these heightened expectations have been shown to increase treatment for all conditions, including those that may be marginally beneficial or even inappropriate. Kravitz et al. reported that standardized patients with adjustment disorder (a condition that is usually treated without medication) who made a request for a brand name antidepressant were five times more likely to receive a prescription for antidepressants, which, in this context, is “at the margin of clinical appropriateness” [ 12]. Heightened expectations may lead to inappropriate and costly demands for medications when evidence would dictate other medications or nonpharmacological interventions.

Optimal use of DTCA may require stricter guidelines on advertisements or more aggressive enforcement of current guidelines so that patients do not form unreasonable expectations. Diminishing the demand for inappropriate prescriptions would lessen the negative impacts of DTCA. Meanwhile, exposure to DTCA might, nonetheless, continue to improve health practices and outcomes through its ability to facilitate favorable clinical responses. By understanding the expectations that DTCA creates, physicians may limit the problems associated with DTCA, while harnessing this placebo effect to increase the effectiveness of prescribed treatment.

## Richard Kravitz's Viewpoint: Regulate, Don't Ban—The Power of DTCA Should Be Harnessed for the Public Good

The opposing positions on DTCA of prescription drugs are well known. Proponents tend to focus on DTCA's potential to educate consumers and encourage productive interchange between patients and physicians, while critics emphasize liabilities. In the US, reasoned discourse has nearly suffocated in an atmosphere thick with First Amendment objections (the First Amendment protects the right to free speech) served up by lawyers for Big Pharma and, on the opposing side, the occasional anticorporate rant.

A more dispassionate analysis would acknowledge three facts. First, prescription drug costs are rising rapidly—based on a confluence of increased prices, increased use of existing drugs, and introduction of new (more expensive) drugs, many of which are promoted directly to the consumer [ 13].

Second, some drugs are clearly overprescribed. (Overprescribing at the level of the individual implies that the benefits of taking the drug do not clearly outweigh the risks.) Overuse has been seen with antibiotics for viral upper respiratory track infections [ 14]; antihistamines, benzodiazepines, and sedative-hypnotics in the elderly [ 15]; inhaled beta-adrenergic agonists in children who are not taking “controller medications” for asthma [ 16]; neuromodulators for chronic pain [ 17]; and sildenafil and fluoxetine for augmentation of normal sexual and psychological functioning, respectively [ 18]. The role of DTCA in promoting such overuse is unclear, although there is little question that advertising lowers the clinical threshold for prescribing.

DTCA is neither good nor evil; it is both.

Third, there is substantial underuse of some prescription drugs in the US. On a population basis, underuse of effective therapies may cause more deaths per year than overuse [ 19]. Examples of underprescribed drugs include beta-blockers following myocardial infarction, angiotensin-converting enzyme inhibitors in congestive heart failure, adjuvant hormonal or chemotherapy following breast cancer surgery, prophylactic antibiotics prior to joint-replacement surgery, and warfarin in patients with atrial fibrillation.

Given that some drugs are underused and others overused, an intervention such as DTCA that increases prescribing could have beneficial effects, deleterious effects, or both. While logically coherent, this conclusion could not until recently claim much empirical support. A recent trial provides strong evidence that DTCA—like the prescription drugs it promotes—has both therapeutic and toxic effects [ 12].

The investigators trained actors (“standardized patients,” [SPs]) to portray patients with major depression of moderate severity (a serious condition requiring treatment, referral, or close follow-up) or with adjustment disorder (a less serious condition in which supportive counseling and watchful waiting might suffice). SPs were randomly assigned to visit 152 primary-care physicians in three US cities (298 visits in all). In one-third of visits, the actresses (all non-Hispanic, white, middle-aged women) mentioned a TV advertisement and made a brand-specific request for Paxil (paroxetine); in another one-third of visits, they made a general request for “medicine that might help”; and in the rest of the visits they made no request.

There were two main findings. First, among SPs presenting with major depression, SPs making no request had only a 56% chance of receiving high-quality initial care (antidepressant prescription, mental health referral, or close follow-up). In contrast, SPs making a brand-specific or general request for medication were treated to this high standard of care in 90% of visits. That's good news, because it suggests that informed, motivated, and involved patients can dramatically improve the quality of their own care. Second, among SPs presenting with adjustment disorder, the proportion receiving an antidepressant prescription was 55% if a brand-specific request was made, 39% if a general request was made, and 10% if no request was made. That's not so good, because it means that requests associated with consumer drug advertisements could lead to lots of prescriptions at the very margins of clinical appropriateness.

In general, DTCA is most likely to deliver public health benefits when the condition to be treated is serious and when the treatment is safe, effective, and underused. DTCA will tend to deliver net harms when the condition is mild or trivial and when the treatment is potentially dangerous, marginally effective, or overused. DTCA—or a social marketing campaign modeled on it—could be an extremely effective way of encouraging patients with a recent myocardial infarction to take aspirin, beta-blockers, or an HMG-CoA-reductase inhibitor (statin). On the other hand, with health-care costs spiraling out of control, it is hard to justify multimillion-dollar advertising campaigns touting drugs for baldness, toenail fungus, and overactive bladder. For obvious reasons, drug companies are not stepping forward with advertisements for the (often generic) medicines that are truly underused.

The question for US policymakers is not whether DTCA should be banned, but how can its benefits be maximized and risks minimized within our free enterprise system. Two policy initiatives hold special promise. A two-year moratorium on DTCA of new drugs, coupled with a requirement for systematic postmarketing surveillance, could avoid another Vioxx tragedy, in which drug marketing got well ahead of the science. In addition, the tax system could be used to create incentives for public–private consortia to produce mass media campaigns aimed at educating patients about common, serious medical conditions, and encouraging them to take evidence-based therapies.

DTCA is neither good nor evil; it is both. A little regulatory ingenuity could harness the enormous power of DTCA or DTCA-like public service announcements to improve the public health.

## Peter Mansfield's Viewpoint: There's a Better Way than DTCA

The collective evidence on DTCA suggests that it may have some benefits, but there is stronger evidence of harms ( http://www.healthyskepticism.org/library/topics/dtca.php) [ 20, 21]. Greater benefit could be gained, with less harm, from publicly funded health information and promotion [ 22].

DTCA is limited to drugs that are profitable to advertise: mostly expensive, new drugs for long-term use for common indications. Such advertising increases premature rapid uptake and overuse of new drugs before flaws, including safety problems, have been discovered and communicated to health professionals [ 21, 23, 24]. Many new drugs are inferior to older treatments, and over two-thirds are no better but are often more expensive [ 25]. Increased use of new drugs stimulated by DTCA can lead to adverse events directly (for example, cardiovascular events associated with COX-2 selective inhibitors, which were heavily advertised to the US public) [ 23, 26, 27] or indirectly, by diverting resources from more cost-effective interventions.

DTCA may have negative economic, social, and political consequences.

DTCA rarely focuses on, and tends to drown out, high-priority public health messages about diet, exercise, addictions, social involvement, equity, pollution, climate change, and appropriate use of older drugs. Older drugs are less profitable to advertise because a share of the sales stimulated goes to generic competition. Consequently, DTCA for any currently advertised drug will become less profitable after expiry of patent protection from competition.When DTCA no longer provides competitive return on investment, it is stopped. Consequently, if there are any benefits from current DTCA (such as stimulating new requests for statins after a myocardial infarction), those benefits will be for a limited time only.

DTCA aims to persuade rather than to inform, and there is evidence that it is effective at persuasion [ 12, 21]. Content analyses of DTCA have found that the information provided is usually flawed and incomplete [ 28–32]. Examples include a study of 320 drug advertisements in popular US magazines that found that the advertisements rarely provided information about success rates of treatment or alternative treatments [ 32], and a study of 23 US television advertisements for prescription drugs that found that the majority gave more time to benefits than to risks [ 28].

Such advertising can lead some people to falsely believe they are well informed, so it reduces their motivation to search for more reliable information. Finding reliable information is already difficult (like finding a needle in a haystack) and the “noise” of DTCA just makes the haystack larger.

Advertising drugs to the public often works by creating or exacerbating unhappiness or anxiety about symptoms or normal experiences (such as occasional erectile difficulties), and by creating high expectations of benefit from drugs. The combination of heightened unhappiness and high expectations can cause severe distress when a drug is unaffordable or when its effects are disappointing: for example, a qualitative study of men who used sildenafil for erectile dysfunction found that expectations raised by media hyperbole had an adverse effect on the morale of those for whom it was ineffective [ 33].

DTCA is often ambiguous and widens the indications beyond those for which the promoted drugs are worthwhile. For example, DTCA may have contributed to increasing unjustified use of antidepressants for young people [ 34, 35].

DTCA may also have negative economic, social, and political consequences. For example, by increasing use of expensive drugs and increasing adverse events [ 21, 23, 24], DTCA increases taxpayer, insurance, and individual costs, which in turn can harm individual, familial, and national economies. The heavy costs of DTCA contribute to higher drug prices and are a hurdle for market entry of new competition. Revenue from DTCA creates a conflict of interest for media companies, because such advertising can undermine the media's freedom to report critically on the drug industry. DTCA can have a distorting effect on people's perceptions of health and disease, including promoting the medicalization of conditions that are within the spectrum of normality [ 36]. DTCA sometimes persuades people to interpret distress as signifying individual illness rather than social or political problems to be solved. DTCA pushes a “Brave New World” where if “anything unpleasant should somehow happen, why, there's always [the sedative] soma to give you a holiday from the facts. And there's always soma to calm your anger, to reconcile you to your enemies, to make you patient and long-suffering” [ 37].

DTCA increases taxpayer, insurance, and individual costs.

There are two root causes of the problems with DTCA. The first is payment systems that reward drug companies for increasing sales of expensive drugs regardless of the impact on health. These systems should be redesigned [ 22, 38]. The second root cause is normal human vulnerability to being mislead [ 39]. Few people have the time and advanced skills in drug evaluation, psychology, logic, economics, and semiotics, etc., required to evaluate drug promotion. Advertising can sneak in under the radar to influence even skeptical people without their awareness. Ideas that would be rejected if given attention get reinforced by repetition. More research is needed to test the hypothesis that it is possible to learn how to gain more benefit than harm when exposed to drug promotion [ 40].

Almost all government, health professional, and consumer inquiries into DTCA have concluded that it causes net public harm [ 20, 41]. It is too difficult to regulate DTCA, so I believe that the logical conclusion from the evidence is that the best option for improving overall health and wealth is to ban all types of DTCA, including “disease awareness” advertising [ 42]. Drug company Web sites and media releases should be regulated carefully.

The public would benefit from reliable information and health promotion focused on public health priorities. Such information can be provided at no extra cost by copying, improving, and expanding policies and programs that are already successful in many countries. Governments and insurance companies who subsidize drugs currently pay for biased promotion indirectly via high drug prices. Instead, these agencies could fund information, education, and promotional services focused on public health needs. Such investments pay for themselves by reducing health-care costs. Universities and nonprofit organizations are well placed to compete for this funding. These organizations are more trustworthy than drug companies because they don't gain from drug sales. Where behavior-change promotion is justified, these organizations could collaborate with advertising agencies. This collaborative approach has already been successful for many health-promotion campaigns—for example, promoting smoking cessation. These improvements would not achieve utopia, but would improve health and increase wealth overall.

## Abbreviations

DTCA: direct-to-consumer advertising
SP: standardized patient
